# ArterioVenous Malformation within Jejunal Diverticulum: An Unusual Cause of Massive Gastrointestinal Bleeding

**DOI:** 10.1155/2009/384506

**Published:** 2009-09-10

**Authors:** Jeffrey K. Lee, John M. Carethers, Pradipta Ghosh

**Affiliations:** Department of Medicine, Division of Gastroenterology, University of California, San Diego, CA 92103, USA

## Abstract

Massive gastrointestinal (GI) bleeding can occur with multiple jejunal diverticulosis. However, significant bleeding in the setting of few diverticulae is very unusual and rare. We report a case of massive gastrointestinal bleeding from an arteriovenous malformation (AVM) within a jejunal diverticulum to underscore the significance of such coexisting pathologies. Mesenteric angiogram was chosen to help identify the source of bleeding and to offer an intervention. Despite endovascular coiling, emergent intestinal resection of the bleeding jejunal segment was warranted to ensure definitive treatment. However several reports have shown jejunal diverticulosis as a rare cause of massive GI bleeding. The coexistence of jejunal diverticulum and AVM is rare and massive bleeding from an acquired Dieulafoy-like AVM within a diverticulum has never previously been described. Awareness of Dieulafoy-like AVM within jejunoileal diverticulosis is useful in preventing delay in treatment.

## 1. Introduction

Jejunal diverticulosis is a rare clinical entity that is often asymptomatic [1]. Massive gastrointestinal (GI) bleeding can occur with multiple jejunal diverticulosis [2]. However, significant bleeding in the setting of few diverticulae is very unusual and rare [1] and should raise the suspicion for secondary coexisting lesions within the diverticulae, for example, ArterioVenous malformations (AVMs), adenomas, or tumors. We report a case of massive gastrointestinal bleeding from an AVM within a jejunal diverticulum to underscore the significance of such coexisting pathologies.

## 2. Case Presentation

**Figure 1:**
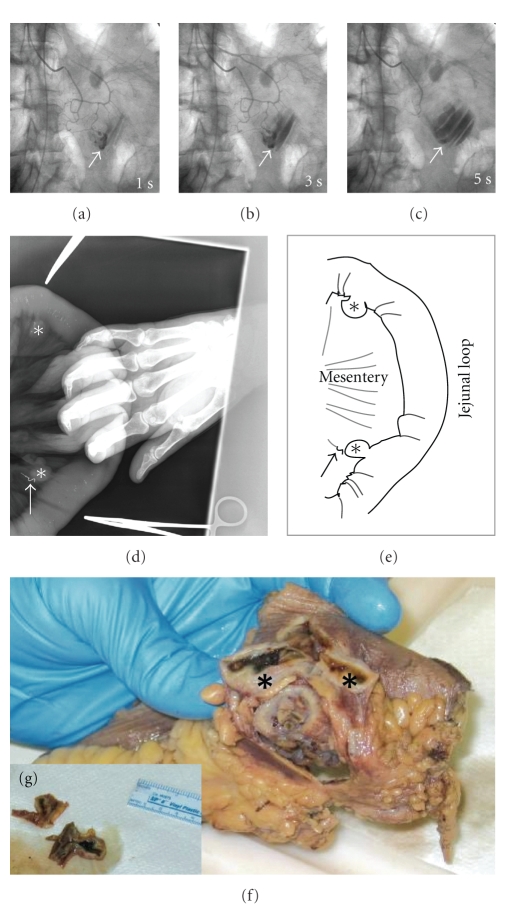
*Massive gastrointestinal bleeding from an ArterioVenous malformation within a jejunal diverticulum*. Superior Mesenteric Angiography (a)–(c) revealed active extravasation (arrow) of the contrast material in the proximal jejunum and pooling of the same (arrow) in the jejunum (c). Intraoperative plain film X-ray obtained during segmental resection of the proximal jejunum (d, e) revealed the presence of two diverticula (stars), located on the mesenteric side. The arrow marks the diverticulum that was the source of hemorrhage, as determined by the presence of the radio opaque angiographic coil which was previously deployed during angiographic intervention. The specimen from segmental jejunal resection (f, g) shows blood clot within the diverticula.

**Figure 2:**
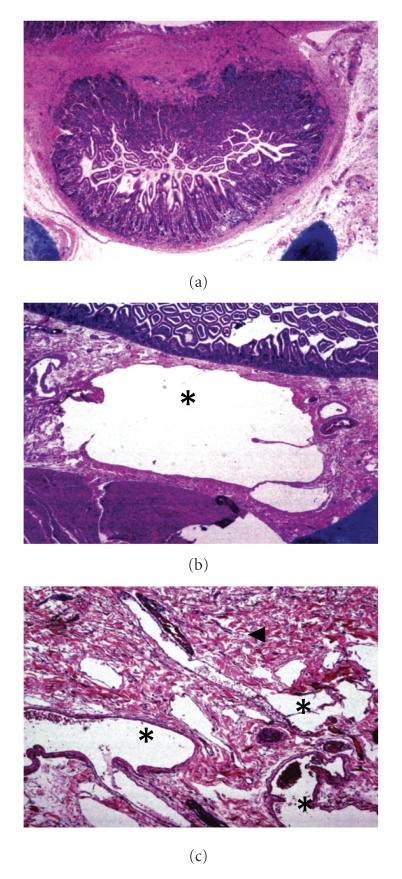
*Histopathologic examination* by a low power microscopy and hematoxylin and eosin staining confirmed that both diverticula were composed of all layers of the intestinal wall: mucosa, muscularis mucosa, and muscularis propria (a), which is characteristic of true diverticula. A thorough examination of sections through the base of the culprit diverticulum revealed the presence of cavernous spaces (stars) lined by endothelium, interspersed by loose connective tissue (arrowhead), but often communicating with each other (b,c), consistent with the presence of submucosal ArterioVenous malformation.

A 91-year-old previously healthy woman presented with hematochezia and hypotension. Esophagogastroduodenoscopy with push enteroscopy and colonoscopy revealed fresh bleeding from an inaccessible segment of small bowel between the mid jejunum and terminal ileum. Selective splanchnic arterial angiography showed extravasation of contrast from the mid-jejunal branch of the superior mesenteric artery (Figures 1(a), 1(b), and 1(c)). Endovascular coiling of the bleeding artery at angiography only achieved short-lived hemostasis, necessitating surgical intervention. Intraoperatively, two jejunal diverticula were identified on the mesenteric border (Figures 1(d) and 1(e)); one of them with previously placed endovascular coil (arrow) implicated as the source of bleeding. A curative resection of the segment of jejunum containing the two diverticula was performed (Figures 1(f) and 1(g)). Histopathologic examination confirmed this as true diverticula (Figure 2(a)) with a submucosal Dieulafoy-like AVM surrounded by normal mucosa as the source of bleeding (Figures 2(b) and 2(c)). Calcified atherosclerosis within the small mesenteric artery feeding this malformation was appreciated, likely contributing towards the pathogenesis of the Dieulafoy-like AVM. The postoperative course was uneventful and the patient remained free of GI bleeding on her 2-month followup. 

## 3. Discussion

First described by Soemmering and Baillie in 1794, jejunal diverticulosis is a rare clinical entity with an overall frequency of about 0.7% [3]. Some studies report the incidence of jejunal diverticulosis ranging from 0.002 to 4.6%, depending on the diagnostic method used [4–6]. Jejunal diverticula are more frequently seen with increasing age, peaking in the sixth and seventh decades of life, and have a slight male predominance [3]. 

Jejunal diverticulosis is characterized by herniation of mucosa and submucosa through the muscular layer of the bowel wall on the mesenteric border of the jejunum. Though the etiology is still unclear, most agree that increased intraluminal pressure leads to its development [7, 8]. Because the diverticular wall lacks a muscularis layer, jejunal diverticula are considered pseudodiverticula as compared to true congenital Meckel's diverticulum [9, 10]. 

Jejunoileal diverticulosis is usually clinically silent until it presents with complications, which include chronic abdominal pain, malabsorption from bacterial overgrowth, diverticulitis, hemorrhage, small bowel obstruction, and perforation [3, 9, 11]. Hemorrhage from jejunal diverticula usually presents as lower GI bleeding; however several cases of hematemesis have been reported [12]. This bleeding may be acute or chronic; however iron deficiency was not noted in our patient. The mechanism of diverticular hemorrhage is similar to that seen in the large bowel in that the diverticulum erodes through a perforating artery. Unlike the large bowel, the stool is usually liquid in the jejunum and propulsion is not associated with high intraluminal pressures and progressive increase in size of small bowel diverticula is infrequent in the absence of motility disorders or luminal obstruction. 

Vascular lesions such as AVMs and venous ectasias are the most common causes of small bowel hemorrhage [13]. However, the coexistence of bleeding AVM within a jejunal diverticulum is rare and to the best of our knowledge, there are only two prior reports claiming such coexistence [14, 15]. However, in both those cases the diagnosis of AVM within a jejunal diverticulum was either not confirmed at pathology [14] or angiographic localization of the bleeding to the angiodysplastic lesion was not performed [15]. In our patient, the angiostatic coil deployed during angiography, thereby serving as a landmark for the exact source of bleed was subsequently visualized at the site of bleeding diverticula during laparotomy. This coil was later retrieved during pathologic examination of the resected bowel from the bleeding diverticula, within the blood vessel that fed the culprit AVM, thereby providing a definitive proof for the source of bleeding. In addition to vascular abnormalities, bleeding from common pathologies of the GI tract (e.g., infarcts, carcinoid, ectopic pancreatic rest, NSAID-related ulcers, adenomas, or adenocarcinomas) can coexist within the diverticula, whose mucosa is as predisposed as any other segment of the bowel. Identification of these secondary etiologies is important because in some cases, this may alter the course of treatment and followup. 

Evaluation of bleeding jejunal diverticula can be identified by radioisotope-tagged red cells, angiography, or double-balloon enteroscopy [16]. Capsule endoscopy has also been used to diagnose jejunal diverticulum; however the risk of retention in the diverticulum has been seen in several cases [17, 18]. The most sensitive imaging studies to detect bleeding jejunal diverticula are the technetium-99m red blood cell scan and mesenteric angiography, with the latter having the advantage of embolization [19]. The pathognomonic angiographic sign of a bleeding diverticulum is extravasation of contrast into the diverticulum, which was seen in our case. 

Treatment options depending on their hemodynamic status include angiography with embolization, double balloon enteroscopy [16, 20], and laparotomy. In our case, mesenteric angiogram was chosen to help identify the source of bleeding and to offer an intervention if needed. Despite endovascular coiling, emergent intestinal resection of the bleeding jejunal segment was warranted to ensure definitive treatment. 

In conclusion, several groups have reported that GI bleeding from jejunal diverticulosis is rare [2, 19] and massive bleed in the setting of few diverticula is further unusual. In these unusual cases, mucosal lesions, for example, AVMs, adenomas, ulcers, and so forth, coexisting within the diverticula may be the primary source of bleeding. Awareness of coexisting pathologies within jejunoileal diverticulosis is therefore useful in preventing delay in treatment.
